# Reducing Periconceptional Methylmercury Exposure: Cost–Utility Analysis for a Proposed Screening Program for Women Planning a Pregnancy in Ontario, Canada

**DOI:** 10.1289/ehp.1409034

**Published:** 2015-05-29

**Authors:** Janet Gaskin, Colin Rennie, Doug Coyle

**Affiliations:** 1 Epidemiology and Community Medicine, and; 2 Civil Engineering, University of Ottawa, Ottawa, Ontario, Canada

## Abstract

**Background:**

The assessment of neurodevelopmental effects in children associated with prenatal methylmercury exposure, from contaminated fish and seafood in the maternal diet, has recently been strengthened by adjustment for the negative confounding resulting from co-exposure to beneficial polyunsaturated fatty acids (PUFAs).

**Objectives:**

We aimed to determine the cost-effectiveness of a periconceptional screening program of blood mercury concentration for women planning to become pregnant in Ontario, Canada. Fish intake recommendations would be provided for those found to have blood mercury levels above the intervention threshold.

**Methods:**

Analysis was conducted using a combined decision tree/Markov model to compare the proposed screening intervention with standard care from a societal perspective over a lifetime horizon. We used the national blood mercury distributions of women 20–49 years of age reported in the Canadian Health Measures Survey from 2009 through 2011 to determine the cognitive deficits associated with prenatal methylmercury exposure for successful planned pregnancies. Outcomes modeled included the loss in quality of life and the remedial education costs. Value of information analysis was conducted to assess the underlying uncertainty around the model results and to identify which parameters contribute most to this uncertainty.

**Results:**

The incremental cost per quality-adjusted life year (QALY) gained for the proposed screening intervention was estimated to be Can$18,051, and the expected value for a willingness to pay of Can$50,000/QALY to be Can$0.61.

**Conclusions:**

Our findings suggest that the proposed periconceptional blood mercury screening program for women planning a pregnancy would be highly cost-effective from a societal perspective. The results of a value of information analysis confirm the robustness of the study’s conclusions.

**Citation:**

Gaskin J, Rennie C, Coyle D. 2015. Reducing periconceptional methylmercury exposure: cost–utility analysis for a proposed screening program for women planning a pregnancy in Ontario, Canada. Environ Health Perspect 123:1337–1344; http://dx.doi.org/10.1289/ehp.1409034

## Introduction

Prenatal mercury (Hg) has been associated with adverse neurodevelopmental effects at exposures detected at levels found in the general public from exposure to methylmercury (MeHg) contaminated fish and seafood in the diet (Grandjean et al. 2003; Karagas et al. 2012; Oken et al. 2008). Subclinical neurotoxic effects, including neurophysiological and neuropsychological effects, associated with population-level Hg exposures were identified from three prospective cohort studies initiated in the 1980s, in the Faroe Islands (Budtz-Jørgensen et al. 2000, 2004; Grandjean et al. 1997, 1998), in New Zealand (Crump et al. 1998), and in the Seychelle Islands (Myers et al. 2003). Recently, the importance of adjusting for negative confounding has been reviewed (Choi et al. 2008); negative confounding results from co-exposure to beneficial polyunsaturated fatty acids (PUFAs) when Hg contaminated fish and seafood is consumed. A strengthened association of prenatal MeHg with adverse neurodevelopmental effects has been reported after adjustment for PUFAs as a covariate in prospective cohort studies in the Faroe Islands (Budtz-Jørgensen et al. 2007), in the United States (Lederman et al. 2008; Oken et al. 2008), and in a second cohort in the Seychelle Islands (Strain et al. 2008). The New Zealand study comprised mothers matched on fish intake, and thus implicitly included adjustment for PUFAs. Adverse neurodevelopment effects were detected at very low Hg exposures in two U.S. prospective cohort studies (Lederman et al. 2008; Oken et al. 2008).

The World Health Organization (Poulin and Gibb 2008) recommends adopting a population health perspective to assess the burden of exposures that can be reduced by intervention strategies. One such burden is prenatal Hg exposure; the increase in mild mental retardation in a population can be calculated from the shift in IQ distribution that results from the cognitive deficits associated with the distribution of Hg exposure in pregnant women, approximated from that in women of child-bearing age.

Several economic analyses have recently been published assessing xenobiotic metal toxicity, both mercury and lead, from a societal perspective. The burden of MeHg neurodevelopmental toxicity has been assessed in terms of IQ loss and the associated lost lifetime earnings in the United States (Rice et al. 2010; Trasande et al. 2005) and in Europe (Bellanger et al. 2013). The assessment of the economic benefits of preventing Hg exposures above three intervention thresholds in Europe (Bellanger et al. 2013) was based on the association of prenatal Hg with cognitive deficits adjusted for co-exposure to PUFAs, and a lower Hg exposure threshold for adverse effects. Roman et al. (2011) recommended development of a dose–response relationship between Hg exposure and cardiovascular risk so that this risk can be included in future population health risk assessments of Hg. An estimate of this risk was included in the economic analysis by Rice et al. (2010). Evaluations of the burden of childhood lead (Pb) exposure predate those of prenatal MeHg exposure and have been more complex, including consequences of IQ loss in outcomes of reduced quality of life and societal costs, such as remedial education costs, and increased criminal justice and health care costs (Glotzer et al. 1995; Muennig 2009). A partial cost–benefit analysis was conducted for a blood Pb screening program for children from a societal perspective in France (Pichery et al. 2011), which included remedial education costs, lost lifetime earnings associated with IQ loss, and criminal justice system costs.

The objective of this economic analysis is to estimate the quality of life to be gained from reducing prenatal Hg exposures, to determine the cost-effectiveness of a periconceptional screening program of blood Hg concentration for women planning to become pregnant in Ontario, Canada, from a societal perspective. The blood Hg distributions [Canadian Health Measures Survey (CHMS) (StatCan 2013)] were used to characterize exposures of women 20–49 years of age, and IQ loss was calculated using the association, adjusted for PUFAs, of cord blood Hg with cognitive deficits.

## Methods

*Study perspective and time horizon*. A societal perspective and a lifetime analytic horizon are appropriate for this study because the neurodevelopment effects of prenatal Hg exposure is thought to be persistent and irreversible (Debes et al. 2006; Murata et al 2007). The outcomes modeled are reduced quality of life, occurring over a lifetime, and remedial education costs, from junior kindergarten through grade 12. Remedial education costs are provided by the Ontario government.

*Intervention strategies*. Two treatment comparators are evaluated for women planning to become pregnant in Ontario: the reference case and the intervention case. The reference case represents the current situation, with no assessment of blood Hg level. The intervention case consists of the proposed blood Hg screening program for women planning to become pregnant, between the ages of 20 and 49 years; the fish consumption advice (Department of Health and Human Services/U.S. Environmental Protection Agency 2004), provided for those with levels above the intervention threshold, recommends eating two servings of fish per week, substituting small oily fish that are high in healthy PUFAs and low in MeHg (e.g., salmon, sole, trout, shrimp) for high-Hg fish (shark, swordfish, fresh tuna). Three intervention thresholds are assessed. The base case uses an intervention threshold for maternal blood of 3.4 μg/L. Two higher intervention thresholds are evaluated in the sensitivity analysis, at 8 μg/L [the Canadian provisional interim blood guidance value (Legrand et al. 2010)], and at 4.7 μg/L (the Canadian provisional value divided by the mean ratio of cord to maternal blood Hg).

**Figure 1:**
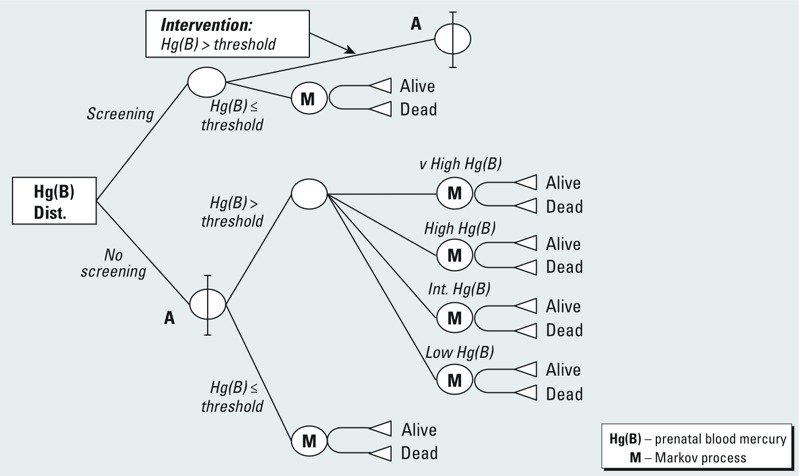
Combined decision tree/Markov model. Dist., distribution.

*Analytical framework*. A combined decision tree/Markov model, shown in Figure 1, is used to analyze the cost-utility of the two treatment comparators. The cohort is followed from the periconceptional period until death. Prenatal Hg exposure will be closely related to the maternal blood Hg levels during the periconceptional period, when the costs of screening and interventions occur, and is represented by the decision tree. The lifelong impacts of mild mental retardation (MMR), defined as having an IQ between 50 and 70, associated with prenatal Hg exposure are calculated from the Markov part of the model using an annual cycle. Two outcomes over the lifetime, alive and dead, are modeled for the cohort using the 2009 all-cause age-specific Ontario mortality rates as the transition probabilities from alive to dead, published in the Death Database [Statistics Canada (StatCan) 2009]. The proportion of children who are shifted from IQ slightly > 70 to < 70 by the cognitive deficits associated with their prenatal Hg exposures is calculated from the maternal blood Hg distribution and the standard normal IQ distribution. The only difference between the two treatment comparators in the model is the intervention. The reference case subtree (labeled A in Figure 1) is repeated in the intervention case after the screening and effect of dietary recommendations. The incremental costs of screening, and diet recommendations when required, are offset by a reduction in remedial education costs and a relative improvement in quality of life from preventing cases of MMR. The results are modeled using an Excel (Microsoft Corporation) spreadsheet, and parameter data used in the model are listed in Table 1.

**Table 1:** Data elements of the economic model.

Data element	Base analysis	Source
Blood Hg distribution		StatCan 2013
Women 20–39 years of age
Geometric mean	0.66 μg/L (95% CI: 0.47, 0.92)
75th percentile	1.6 μg/L (95% CI: 1.1, 2.1)
Women 40–59 years of age
Geometric mean	0.82 μg/L (95% CI: 0.65, 1.0)
75th percentile	1.7 μg/L (95% CI: 1.1 2.2)
90th percentile	2.9 μg/L (95% CI: 1.9, 3.9)
Ratio cord to maternal blood Hg	1.7	Stern and Smith 2003
Weighted average of standard deviation of mercury effect on neurobehavioral test from true prenatal exposure doubling	9.36%	Budtz-Jørgensen et al. 2007
Intervention		Kim et al. 2006
Probability of successful intervention	0.857
Effectiveness of successful intervention	0.556
Effectiveness of unsuccessful intervention	0
Live birth rate by maternal age (years)^*a*^		StatCan 2011
20–24	0.122
25–29	0.280
30–34	0.345
35–39	0.181
40–44	0.039
45–49	0.003
Infertility rate by maternal age (years)^*b*^		Bushnik et al. 2012
18–24	0.070
25–29	0.085
30–34	0.108
35–39	0.130
40–44	0.143
Screening costs
Laboratory blood Hg test	Can$23.27	MOHLTC 2012
Family doctor, intermediate appointment	Can$33.70	MOHLTC 2013
Remedial education SEPPA costs		OME 2012
JK–grade 4	Can$931.29
Grades 5–8	Can$715.34
Grades 9–12	Can$472.92
QALY disutility for MMR (EQ5D)	–0.38	Oostenbrink et al. 2002
Discount rate	5%	CADTH 2006
Abbreviations: EQ5D, EuroQuol-5D, European quality of life–5 dimensions; MMR, mild mental retardation (IQ between 50 and 70); QALY, quality-adjusted life year; SEPPA, special education per pupil amount. Disutility is defined as the loss in utility. ^***a***^The proportion of live births for a given age range out of the total live births over all maternal ages. ^***b***^The proportion of co-habiting couples who did not become pregnant after exposure to the risk of conception during the previous 12 months.		

*Discounting*. The discount rate used for the base analysis is 5%, as recommended by the Canadian Agency for Drugs and Technologies in Health (CADTH 2006). Sensitivity analyses are conducted using a discount rate of 0, 3%, and a variable discount rate, decreasing from 5% for the first 10 years to 3% for 5 years to 1% thereafter.

*Distribution of maternal blood Hg*. Total blood Hg level is the biomarker for prenatal exposure available in Canada [Canadian Health Measure Survey (CHMS) (StatCan 2013)]. MeHg has a relatively long half-life, about 70 days, and so comprises 90% of the total blood Hg for the general public, with most resulting from eating Hg-contaminated fish and seafood (Grandjean et al. 2003; Oken et al. 2008). The CHMS is a national survey that represents 96% of the population 3–79 years of age, and the prenatal exposure for women 20–49 years of age was determined from the national blood Hg distributions for women 20–39 and 40–59 years, extrapolated to Ontario due to limited data. Reliable estimates were reported for geometric mean of 0.66 μg/L [95% confidence interval (CI): 0.47, 0.92] and 75th percentile of 1.6 μg/L (95% CI: 1.1, 2.1) for women 20–39 years, and geometric mean of 0.82 μg/L (95% CI: 0.65, 1.0) and 90th percentile of 2.9 μg/L (95% CI: 1.9, 3.9) for women 40–59 years of age (Table 1). The survey has a cluster design and estimates are subject to sampling error; those estimates with a coefficient of variation between 0 and 16.6% were considered reliable.

There is no evidence of a threshold value for prenatal Hg below which there are no adverse effects; a linear dose–response model describes small adverse effects even at low doses. However, the calculation of the benchmark dose (BMD) and the lower limit of its 95% confidence interval (BMDL) has been widely adopted to provide an approximate threshold level that Budtz-Jørgensen et al. (2000) state “can be interpreted as a No-Observed Adverse Effect level (NOAEL) from experimental studies (Crump 1995).” The BMDL has been found to depend highly on the dose–response model selected; lower BMDLs result from using a logarithmic model for cognitive deficits associated with cord blood Hg than when using a linear model (Budtz-Jørgensen et al. 2001). Observational data used to determine BMDs and BMDLs are based on exposures that are seldom measured without error. Budtz-Jørgensen et al. (2004) showed that if this measurement error is ignored, then the BMDLs produced by the benchmark approach are biased toward higher and less protective levels.

In this analysis we excluded the smallest effects by using a model threshold for adverse effects of 5.8 μg Hg/L in cord blood. This value corresponds to the lower benchmark dose (BMDL) divided by the composite uncertainty factor of 10 used to derive the reference dose assuming a linear dose–response relationship by the National Research Council (2000). The average of the BMDLs using a logarithmic dose–response model for the measure of neurodevelopment most sensitive to Hg was similar at about 5 μg/L in cord blood (Budtz-Jørgensen et al. 2000). The mean ratio of cord to maternal blood Hg concentration used in this analysis was 1.7 (95% CI: 1.0, 3.4), reported in a meta-analysis of 10 studies (Stern and Smith 2003) and used in a burden of disease analysis by Trasande et al. (2005).

Five categories of cord blood Hg concentration are used in this analysis: below the model’s threshold for adverse cognitive effects, and four categories above it: low, intermediate, high, and very high. The cord blood Hg [Hg(Bcord)] categories are defined in units of micrograms Hg per liter: Hg(Bcord) ≤ 5.8, 5.8 < Hg(Bcord) ≤ 7.3, 7.3 < Hg(Bcord) ≤ 10.2, 10.2 < Hg(Bcord) ≤ 13.0, and Hg(Bcord) > 13.0. A probability distribution is used to describe the ratio of cord to maternal blood Hg explicitly, so the maternal blood Hg categories are defined as the cord blood Hg concentration divided by the ratio of cord to maternal blood Hg. For example, the five categories of maternal blood Hg concentration [Hg(Bmat)] calculated using the mean ratio of 1.7 for cord to maternal blood Hg, in micrograms Hg per liter, are Hg(Bmat) ≤ 3.4, 3.4 < Hg(Bmat) ≤ 4.3, 4.3 < Hg(Bmat) ≤ 6.0, 6.0 < Hg(Bmat) ≤ 7.7, and Hg(Bmat) > 7.7.

*Cognitive deficits associated with cord blood Hg concentration*. The association between cord blood Hg and cognitive deficits determined by Budtz-Jørgensen et al. (2007) was used in this analysis because this was the only study that adjusted for negative confounding from co-exposure to PUFAs, assuming a 100% precision in assessment of fish intake, and used structural methods to account for measurement error in the exposure indicators and clinical outcomes. Structural equation models (SEM) include an error term for each measured exposure and outcome, and can be used to model the association between the true exposure and the true outcome.

The Hg effects on five statistically significant neurobehavioral outcome functions, for a doubling in true Hg exposure, were presented in percentage of the standard deviation (Budtz-Jørgensen et al. 2007): motor and verbal at 7 years, and motor, attention, and verbal, at 14 years, at –12.2, –10.8, –9.37, –9.54, and –6.87% SD, respectively. These values are weighted according to the estimated relevance to cognition quantified as IQ points by Cohen et al. (2005), at 0.2 for motor domain, 0.6 for verbal domain, and 0.3 for attention domain. This results in an effect of 9.4% of SD in IQ distribution (SE = 0.93%), equal to 1.4 IQ points (0.094 × 15 points = 1.4 points) for a doubling of prenatal Hg exposure. This was the value used in Bellanger et al. (2013), the only published economic analysis to include adjustment for negative confounding from PUFAs.

Bias can result from the choice of dose–response model used in an analysis; more relative importance to outliers results from the use of a linear relationship, and more relative importance to moderate values results from the use of a logarithmic relationship. Because cost–utility values are used to determine public health priorities, an estimate that is conservative regarding the expected benefit of reducing prenatal Hg exposures was deemed appropriate. This model conservatively used a linear dose–response relationship for the moderate Hg exposures, to give a lower bound for the expected benefit of reducing these moderate exposures, and a logarithmic dose–response relationship for the highest values. The average IQ losses corresponding to low, medium, high, and very high maternal blood Hg are: 0.125, 0.5, 1, and 1.25, respectively, of the 9.4% of a standard deviation, or 1.4 IQ pts, associated with doubling prenatal Hg. The probabilistic values of these IQ losses are modeled by a gamma probability distribution with shape and scale values (101, 0.000926), from the mean of 9.4% (SE = 0.93%) of a standard deviation (15 IQ pts).

*Estimated effect of intervention on maternal blood Hg concentration*. The probability and the effectiveness of the intervention in reducing the blood Hg levels were derived from the only study published of a similar intervention, a small clinical trial in South Korea (Kim et al. 2006). Maternal screening was conducted in early pregnancy, and the intervention was a recommendation to avoid eating fish. The initial and final maternal blood Hg concentrations were read off the graph for the seven women in the intervention group and the five women in the control group with initial blood Hg > 3.4 μg/L. The intervention was successful at reducing the blood Hg to < 3.4 μg/L for six of those seven women, modeled as a beta distribution with shape and scale of (6, 1). Its effectiveness was a mean decrease in blood Hg (relative to the initial value) of 0.56 (SE = 0.11) for a successful intervention, modeled as a beta distribution with shape and scale of (11.6, 9.2).

*Estimated incremental increase in MMR from prenatal Hg exposure*. This analysis adopted the population health approach recommended by the World Health Organization to quantify the burden of neurodevelopmental toxicity of methylmercury as the “rate of mild mental retardation caused by methylmercury-related IQ loss” and the concommitant loss in quality of life calculated from the methylmercury exposure distribution of the population (Poulin and Gibb 2008). Although there is no clear individual burden from the small subclinical IQ deficits that are most common, these small IQ deficits can be significant in a population because they can shift the entire IQ distribution. The individuals who are most heavily affected are children with IQ scores just at the upper IQ threshold for MMR, for whom a lowered IQ score would result in a designation of MMR. It was recommended that the burden of developmental neurotoxicity be quantified.

Each MeHg exposure is associated with a specific IQ loss, determined using the standard normal IQ distribution. The proportion of children shifted into the category of MMR (IQ 50–70) are those who have an IQ just above 70 before it is adjusted for the loss in IQ associated with their prenatal exposure, for whom the adjustment results in an IQ lower than 70. The proportion of potential mothers at each defined blood Hg range is multiplied by the proportion of children who would be shifted into MMR by the specific IQ loss associated with that prenatal Hg exposure, and the value ranges are summed over the blood Hg ranges. This sum is multiplied by the number of live births at each maternal age range, and summed to give the total number of MMR cases that could be prevented. The age-specific infertility rates for women (Bushnik et al. 2012), derived from the 2009–2010 cycle of the Canadian Community Health Survey, were used to adjust the 2011 age-specific Ontario live births reported in the Birth Database (StatCan 2011). Ninety percent of pregnancies planned by women 20–49 years of age result in live births.

*Quality of life*. Quality-adjusted life years (QALYs) are used to estimate the modifiable burden of cognitive deficits from prenatal Hg exposure. Health-related quality of life can be characterized using a questionnaire, such as the EuroQol-5D (EQ5D) or the Health Utility Index Mark 3 (HUI3), which assess the severity of five and eight dimensions, respectively, and are preference-based, with a value of 1 for perfect health and 0 for death (CADTH 2006). The age-specific QALY values [Canadian Community Health Survey (CCHS) 2009–2010] are used to characterize the health-related quality of life of people having no change in cognitive category from prenatal mercury exposure. Disutility is defined as the loss in health-related utility associated with a single health state or disease, and the disutility for MMR (–0.38, sd 0.021) for EQ5D was derived from a study of postmeningitis MMR (Oostenbrink et al. 2002).

*Estimated costs*. According to a societal perspective, costs include those for the health care sector, screening all women planning to become pregnant and dietary advice for those with blood Hg levels over the threshold; and for the education sector, the remedial education for the children shifted into MMR (IQ < 70) by cognitive deficits due to prenatal Hg exposure. All costs noted in this paper are in Canadian dollars. The incremental cost of screening is limited to the laboratory test (women with a family doctor are assumed to have regular appointments), at $23.27 fee for the blood Hg test [Ministry of Health and Long-Term Care (MOHLTC) 2012]. An intermediate family doctor appointment, $33.70 (MOHLTC 2015), is also included in the screening cost for orphan patients, the roughly 8% of Ontario residents who have no family doctor. The intervention cost is $33.70 for the intermediate appointment at which the family doctor provides fish consumption recommendations. The remedial education costs are conservatively limited to the Ontario Ministry of Education annual special education per-pupil amount (SEPPA) from junior kindergarten (JK) to grade 3, grades 4–8, and grades 9–12 are $931.29, $715.34 and $472.92, respectively [Ontario Ministry of Education (OME) 2012].

*Analysis*. The model calculates the reductions in maternal blood Hg levels achieved by the intervention for maternal blood Hg values above the intervention threshold, whereas IQ losses are calculated according to the model threshold for adverse effects. The analysis presents the total estimated costs and QALYs associated with both treatment comparators per live birth in Ontario, and the increment cost per QALY gained (incremental cost–utility ratio; ICUR) for the proposed periconceptional blood Hg screening program. The data elements of the base case analysis are presented in Table 1, and the results of the analysis using different intervention thresholds are presented in Table 2.

**Table 2:** Estimated costs and QALYs associated with maternal blood Hg screening and interventions versus reference case.

Analysis for discount rate of 5%	Reference case	Screening and intervention at Hg(Bcord)		
		3.4 μg/L	4.7 μg/L	8 μg/L
Costs (Can$)	$2.33	$33.79	$33.82	$34.48
QALYs	18.8676	18.8694	18.8693	18.8686
Incremental costs versus reference case		$31.46	$31.49	$32.14
Incremental QALY versus reference case		0.001743	0.001707	0.000945
Incremental cost per QALY gained versus reference case		$18,051	$18,451	$34,017
Abbreviations: Hg(Bcord), cord blood mercury concentration; QALY, quality-adjusted life year. The model threshold for adverse effects used in the analysis was 3.4 μg/L for all intervention thresholds investigated (3.4, 4.7, and 8 μg/L).				

*Sensitivity analysis*. A univariate sensitivity analysis is conducted for changes from the base case relating to discount rate, QALY disutilities for MMR of –0.56 and –0.76 based on HUI3 A and B evaluations (Oostenbrink et al. 2002), cognitive deficits associated with prenatal Hg assuming a 68% precision in PUFA exposure, and inclusion of cost of a family doctor’s appointment for every screening case. The base case assumes that the measurement of PUFA exposure has no error, that is, 100% precision. Because that is unlikely, the sensitivity of the ICUR is evaluated by considering the scenario where the fish intake questionnaire has only a 68% precision for assessing PUFA exposure, for which motor function at 7 and 14 years were reported to be statistically significant: –13.7 and –10.7, respectively, by Budtz-Jørgensen et al. (2007). The ratio of weighted effects from Table 2 in Budtz-Jørgensen et al. (2007) was used to determine an effect of 10.6% of SD in IQ distribution for a doubling of prenatal mercury exposure, assuming 68% precision in PUFA intake. The intervention threshold used in the base case is the same as the model threshold for adverse effects, whereas the intervention thesholds evaluated in the sensitivity analysis, at 4.7 μg/L and 8 μg/L, are higher than the threshold for adverse effects of 3.4 μg/L (Table 2).

We conducted a probabilistic sensitivity analysis using a Monte Carlo simulation with 5,000 replications. Probability distributions for nine model parameters were incorporated into the analysis: the mean and the standard deviation of the lognormal distribution for blood Hg, for women 20–39 and 40–59 years of age, the ratio of cord to maternal blood Hg, the percentage of a standard deviation in cognitive deficits effected by a doubling of prenatal Hg exposure, the probability and effectiveness of successful intervention, and the disutility associated with MMR. Estimates of incremental costs and QALYs are obtained, with 90% confidence intervals, by sampling from the probability distributions over 5,000 replications. A cost-effectiveness acceptability curve (CEAC) presents the probability that the intervention would be optimal for a range of values of willingness to pay for an additional QALY, for the base discount rate of 5%. Estimates of ICUR and incremental net benefit (INB) are obtained, with 90% confidence intervals.

Underlying uncertainty within the economic analysis is assessed from a value of information analysis. The expected value of perfect information (EVPI) measures the reduction in opportunity loss associated with obtaining perfect information for all parameter values, across a range of values for the willingness to pay for an additional QALY. The partial EVPIs (EVPPIs) provide the opportunity loss associated with each parameter, estimated using the quadrature method (Coyle and Oakley 2008).

## Results

*Base analysis*. The estimated cost of the proposed screening intervention—the base case that has an intervention threshold equal to the threshold for adverse effects—is $33.79 per live birth from a planned pregnancy, whereas the estimated cost of the reference case is $2.33. Table 2 demonstrates that the proposed screening program, using the intervention threshold equal to the threshold for adverse effects of 3.4 μg/L for maternal blood, dominates those using higher intervention thresholds, because the proposed screening program has lower incremental costs and higher incremental QALYs. Therefore, the proposed screening intervention, which has an estimated incremental cost per QALY gained of $18,051, is the most affordable intervention to implement as well as the one offering the most benefit per unit cost.

*Univariate sensitivity analysis*. The sensitivity of the deterministic ICUR for the screening intervention is presented in Table 3. The results of the univariate sensitivity analysis demonstrate that the findings of the base analysis are robust; the ICUR was lower than the base case in all but one scenario, in which the screening requires a family doctor appointment for all women planning pregnancies, with an ICUR of $35,900.

**Table 3:** Sensitivity analysis.

Analysis	Estimated incremental cost per QALY (Can$)
Univariate sensitivity analysis scenario
Base case	$18,051
Discount rate of 3%	$11,859
Discount rate of 0%	$4,453
Variable discount rate	$10,119
HUI3 disutility for MMR of –0.56	$12,249
HUI3 disutility for MMR of –0.76	$9,026
Precision of 68% in fish intake assessment for mercury effect on neurobehavioural tests	$15,660
Initial family doctor appointment needed for each Hg(B) screening	$35,900
Probabilistic sensitivity analysis
Average incremental cost per QALY (ICUR)	$18,209
ICUR, 5th percentile	$9,339
ICUR, 95th percentile	$56,555
Incremental net benefit gained versus reference case, for Can$20,000/QALY	$3.10
INB, 5th percentile	–$20.01
INB, 95th percentile	$36.53
Incremental net benefit gained versus reference case, for Can$50,000/QALY	$54.96
INB, 5th percentile	–$3.56
INB, 95th percentile	$139.08
Abbreviations: Hg(B), blood mercury concentration; HUI3, Health Utilities Index Mark 3; ICUR, incremental cost-utility index (i.e., incremental cost per QALY); INB, incremental net benefit; MMR, mild mental retardation (IQ between 50 and 70); QALY, quality-adjusted life year. The HUI3 disutility for MMR represents the loss in utility associated with having mild mental retardation assessed using the HUI3 questionnaire.	

*Probabilistic sensitivity analysis*. Also in Table 3, the estimated ICUR from the probabilistic sensitivity analysis using a Monte Carlo simulation is $18,209, with a 5th-percentile ICUR of $9,339 and a 95th-percentile ICUR of $56,555. The INB of the screening intervention for a willingness to pay of $50,000 per QALY is $3.10 (90% CI: –$20.01, $36.53).

**Figure 2:**
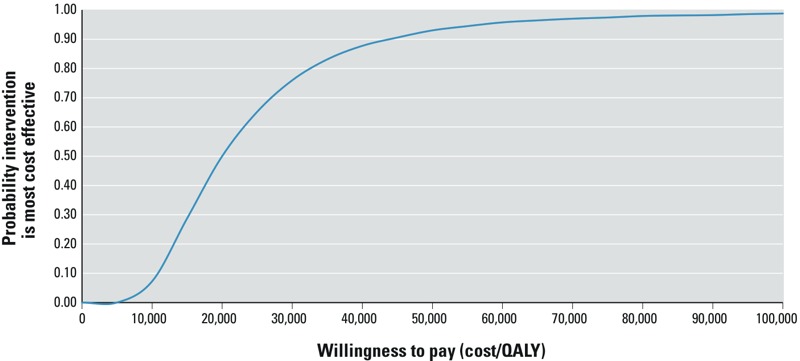
Cost-effectiveness acceptability curve. The cost-effectiveness acceptability curve (CEAC) was derived by plotting the incremental net benefit (INB) for 5,000 Monte Carlo simulations for each of a range of willingness to pay per QALY (Can$). The analysis was based on the model assumptions of the base case analysis (Table 1).

The CEAC (Figure 2) represents the probability that the intervention will be cost-effective over a range of values of willingness to pay per QALY gained. The CEAC is used to aid decision making by showing the probability that funding the intervention would be cost-effective given the uncertainty associated with the parameters used in the model. The CEAC for the proposed screening intervention shows that it would be cost-effective 75% of the time at a willingness to pay of $30,000/QALY and 93% of the time at $50,000/QALY.

**Figure 3:**
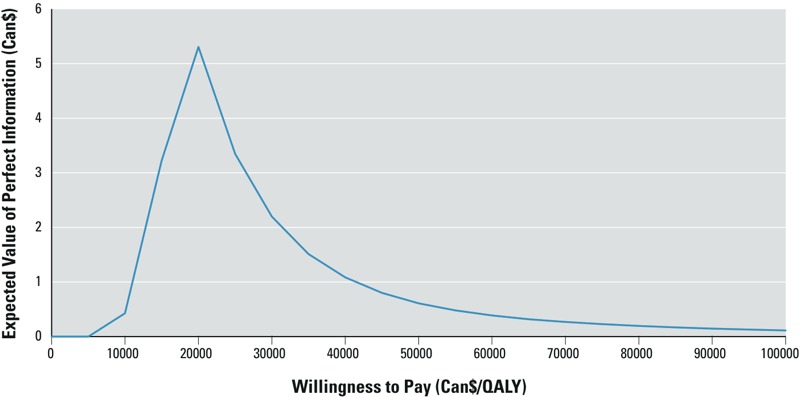
Expected value of perfect information (EVPI) by willingness to pay. The EVPI is derived by calculating, for each value of willingness to pay for a QALY, the mean of the 5,000 Monte Carlo simulations of the difference between the maximum incremental net benefit (INB) of the treatment comparators and the INB of the treatment comparator with the best overall ICUR. The analysis was based on the model assumptions of the base case analysis (Table 1).

The graph of the EVPI by willingness to pay (Figure 3) rises sharply from zero and peaks near $20,000/QALY at about $5 and declines rapidly to < $0.61 at $50,000/QALY.

Table 4 presents the EVPI and the EVPPIs for each of the model parameters. At $50,000 per QALY, the EVPI is $0.61 and parameter with the highest EVPPI is the standard deviation of the blood Hg distribution of women 20–39 years of age ($0.0463) followed by the probability of a successful intervention (< $0.00509).

**Table 4:** Value of information analysis for screening intervention versus reference case.

Value of information	WTP per QALY (Can$)		
	$20,000	$30,000	$50,000
EVPI (Can$)	5.30	2.20	0.61
EVPPI (Can$)
Mean Hg(B) of women 20–39 years	1.41	0.02960	5.77E-06
Standard deviation Hg(B) of women 20–39 years	2.08	0.393	0.0463
Mean Hg(B) of women 40–49 years	0	0	0
Standard deviation Hg(B) of women 40–49 years	0	0	0
Ratio of cord/maternal Hg(B)	2.39	0.0606	0
Percent of a standard deviation in cognitive deficits	0.444	1.17E-05	0
Probability of a successful intervention	1.08	0.102	0.00509
Effectiveness of a successful intervention	1.24	0.0590	6.12E-04
Disutility for MMR	0.0404	0	0
Abbreviations: EVPI, expected value of perfect information; EVPPI, expected value of partial perfect information; Hg(B), blood mercury concentration; MMR, mild mental retardation (IQ between 50 and 70); QALY, quality-adjusted life year; WTP, willingness to pay. Percent of a standard deviation in cognitive deficits is used in the model used to quantify the loss in IQ points associated with a doubling of true prenatal mercury exposure. Disutility is defined as the loss in utility.			

## Discussion

Ontario had 140,135 live births in 2011: 130,045 (92.8%) born to mothers 20–39 years, and 5,886 (4.2%) born to mothers 40–49 years of age. Of these, we estimate that 14,562 (10.4%) would have been born to women with maternal blood Hg > 3.4 μg/L. Very similar Hg exposure is reported for U.S. women of childbearing age in NHANES (National Health and Nutrition Examination Survey, 1999–2003), with 10% of women 15–45 years having blood Hg > 3.5 μg/L (Rice et al. 2010). In North America, roughly 50% of pregnancies are planned (Finer and Zolna 2011), and the Canadian rates are assumed to be very similar. Adjusting for infertility rates, we estimated that 8,084 planned pregnancies would have resulted in the planned live births (in Ontario in 2011) having maternal blood Hg levels > 3.4 μg/L, which could be reduced by the proposed screening and intervention program.

Fish consumption offers clear benefits, with serum PUFAs correlating with cognitive development in two prospective cohort studies (Lederman et al. 2008; Oken et al. 2008). However, these studies also reported adverse cognitive effects associated with prenatal Hg exposures previously considered safe. Fish is the main source of protein in many parts of the world, and fish consumption advice must balance the risk of adverse health effects of mercury with the benefits of essential micronutrients, though controversy remains about just what constitutes the best balance between these dietary co-exposures (Budtz-Jørgensen et al. 2007; Ginsberg and Toal 2009; Karagas et al. 2012). A screening program for periconceptional blood Hg level would support the promotion of healthy fish consumption by reducing anxiety and potential elimination of fish from the maternal diet.

The probabilistic sensitivity analysis estimates the ICUR for the proposed screening intervention at $18,209/QALY (90% CI: $9,339/QALY, $56,555/QALY). Reducing cognitive deficits associated with prenatal Hg exposure, by reducing blood Hg level in women 20–49 years of age planning pregnancies, would be expected to be very cost-effective in Ontario for the base case screening program proposed. Individual benefit would vary widely for this screening program: It is expected to prevent MMR in a few, to prevent a subclinical IQ loss in many, and to provide some peace of mind for most. Although the average incremental QALY gain versus reference case would be very low, at 0.0017 (Table 2), the benefit would be substantial for individuals in whom MMR, associated with a QALY disutility of –0.38, could be prevented by screening for prenatal Hg exposure. A global treaty to protect human health and the environment from the adverse effects of mercury was adopted on 19 January 2013—the UNEP (United Nations Environment Programme) Minamata Convention on Mercury (UNEP 2015)—and this analysis demonstrates how cost-effective this screening intervention could be for reducing prenatal Hg exposures even when using conservative estimates of the potential health benefits.

A conservative approach was adopted for this analysis, which is limited to adverse neurodevelopmental effects of Hg associated with prenatal exposure because a dose–response model has not yet been developed to quantify the cardiovascular risk factors associated with Hg exposure (Murata et al. 2007). The results of the univariate sensitivity analysis show that, if our assumptions are valid, the proposed screening intervention would be more cost-effective for all alternative scenarios considered except when the cost of a family doctor’s appointment for every screening case instead of just for the orphan patients is included. Because the prevention of loss in quality of life would accrue over a lifetime and the prevented remedial education costs would accrue until 17 years of age, whereas the costs of the intervention would occur during the first year, the intervention would be more cost-effective at lower discount rates. The lowest MMR disutility value published, of –0.38 (SE = 0.021) for EQ5D, is used in this analysis (Oostenbrink et al. 2002). We estimate that the intervention would be more cost-effective for the assessments of larger disutility, with ICURs of $12,249/QALY and $9,026/QALY calculated for the HUI3A disutility, –0.56 (SE = 0.026), and the HUI3B disutility, –0.76 (SE = 0.034), respectively. This analysis assumed a 100% precision in fish intake from the questionnaire, whereas a 13% greater adverse effect of prenatal Hg exposure is derived assuming 68% precision (based on the average of effects on motor function at 7 and at 14 years) resulting in an ICUR of $15,660/QALY. The only scenario where the ICUR is higher than the base case was derived from assuming that each screening would require a family doctor’s appointment (ICUR of $35,900).

The most important limitation of this analysis is the determination of the effectiveness and the probability of a successful intervention from a single, very small clinical trial in South Korea (Kim et al. 2006). The proposed intervention would recommend substituting low-Hg/high-PUFA fish for high-Hg fish, rather than eliminating fish altogether, as was recommended in the South Korean trial. Korean cuisine is traditionally high in fish, and although the South Korean trial intervention required a greater change in individual behavior, it was still very effective. Only seven women had an initial blood Hg > 3.4 μg/L in the Korean trial, a very small sample, and some inaccuracies in blood Hg levels could have resulted from reading the values off the graph. Another data limitation is the small sample size from which the maternal blood Hg distribution was derived. The CHMS used a cluster design and sampled 500 people in each sex–age category. This uncertainty is significant because this analysis focused on the elevated levels at the tail of the blood Hg distribution.

The opportunity cost of making a decision under uncertainty is determined from the uncertainty surrounding the cost of the intervention and the value of the outcomes. The value of information analysis suggests that the conclusions concerning the cost-effectiveness of the proposed blood Hg screening program for women planning to become pregnant in Ontario are robust. The estimated expected value of perfect information shows that the opportunity cost could be reduced by reducing the uncertainty of some parameters; and at a threshold value for a QALY of $50,000, the model parameters that contribute most to the uncertainty in the ICUR are the standard deviation of the blood Hg distribution of women 20–39 years of age and the effectiveness of the intervention. It would be feasible to collect further data on the effectiveness of the intervention to reduce the uncertainty in the ICUR, by conducting a clinical trial of the proposed intervention in Ontario. This would be especially informative because the values for effectiveness of the proposed intervention used in the analysis were derived from the intervention in the South Korean trial.

## Conclusions

Our estimates suggest that the proposed periconceptional screening intervention for blood Hg concentration would be highly cost-effective from a societal perspective. The estimated incremental cost–utility ratio is $18,051 per QALY gained, with a probabilistic estimate of $18,209/QALY (90% CI: $9,339/QALY, $56,555/QALY). The estimates for the expected value of partial perfect information suggest that there would be value in obtaining further information to reduce uncertainty about the probability and effectiveness of implementing the proposed intervention in Ontario.
